# The Activity of Carbohydrate-Degrading Enzymes in the Development of Brood and Newly Emerged workers and Drones of the Carniolan Honeybee, *Apis mellifera carnica*

**DOI:** 10.1673/031.012.2201

**Published:** 2012-02-09

**Authors:** Krystyna Żółtowska, Zbigniew Lipiński, Elżbieta Łopieńska-Biernat, Marek Farjan, Małgorzata Dmitryjuk

**Affiliations:** ^1^ Biochemistry Department, Faculty of Biology, University of Warmia and Mazury, Oczapowskiego 1A Str., 10-719 Olsztyn, Poland; ^2^ Veterinary Diagnostic Laboratory BIOLAB, Grunwaldzka 62 Str., 14-100 Ostróda, Poland

**Keywords:** α-glucosidases, amylase, carbohydrate metabolism, disaccharidases, glycogen Phosphorylase, sucrase, trehalase, Western honeybee development

## Abstract

The activity of glycogen Phosphorylase and carbohydrate hydrolyzing enzymes α-amylase, glucoamylase, trehalase, and sucrase was studied in the development of the Carniolan honey bee, *Apis mellifera carnica* Pollman (Hymenoptera: Apidae), from newly hatched larva to freshly emerged imago of worker and drone. Phosphorolytic degradation of glycogen was significantly stronger than hydrolytic degradation in all developmental stages. Developmental profiles of hydrolase activity were similar in both sexes of brood; high activity was found in unsealed larvae, the lowest in prepupae followed by an increase in enzymatic activity. Especially intensive increases in activity occurred in the last stage of pupae and newly emerged imago. Besides α-amylase, the activities of other enzymes were higher in drone than in worker broods. Among drones, activity of glucoamylase was particularly high, ranging from around three times higher in the youngest larvae to 13 times higher in the oldest pupae. This confirms earlier suggestions about higher rates of metabolism in drone broods than in worker broods.

## Introduction

In insects, the fat body is the main organ responsible for energetic metabolism, executing a function similar to that of the liver in vertebrates. This is also the organ of conversion and storage of fat, carbohydrates, and proteins (Arrese and Soulages 2010). The fat body is responsible for metabolism of carbohydrates and is especially enlarged in insect larvae. In honeybee larvae, it can constitute up to 65% of an individual's body mass (Bishop 1923, 1925). During metamorphosis the fat body tissue is very rapidly disintegrated, and products of this process are used to build pupae organs (Bishop 1923). Hormones such as octopamine, juvenile hormone III adipokinetic/hypotrehalosaemic (Mas-AKH), and insulin-like growth factors are the main factors responsible for metabolism within the fat body in insects (Hagenguth and Rembold 1978; Rachinsky et al. 2000; Roeder 2005; Ament et al. 2008).

In insects, the hypotrehalosaemic hormone stimulates degradation of glycogen and synthesis of trehalose in the fat body (Gäde 1999; Lorenz et al. 1999). Likewise in other animals, the neuropeptide results in an increase of fructose-2,6-bisphosphate concentration, a compound responsible for the rate of glycolysis (Becker et al. 1996; Meyer-Fernandes et al. 2001). This hormone was detected in the honeybee strain *Apis mellifera ligustica,* but it was surprising that the activity of Mas-AKH was missing in the corpora cardiaca in all sexes of adult *Apis mellifera carnica* Pollman (Hymenoptera: Apidae) (Lorenz et al. 1999; Woodring et al. 2003). Blatt and Roces (2001) demonstrated that high concentrations (30–50%) of sucrose in food of foraging bees of *A. m. carnica* affect homeostasis of sugars in their hemolymph. The mechanism responsible for regulation of carbohydrate metabolism in larvae of this honeybee strain is still unknown.

The diet of brood and adult honeybees is rich in sugars. The bees gain carbohydrates mainly from nectar and honey, and additionally from pollen in small amounts. Therefore, their energetic metabolism is based generally on carbohydrates. Lipids and amino acids are less important for them as a source of energy (Hepburn et al. 1979; Leta et al. 1996).

Sugars are degraded into glucose and fructose by digestive enzymes in the alimentary tract. The carbohydrate economy of adult bees is well understood, especially the distribution and sugar concentration in tissues (Panzenböck and Crailsheim 1997) and hormonal regulation of sugar metabolism (Woodring et al. 2003; Becker et al. 1996; Gäde 1999; Lorenz et al. 1999; Ament et al. 2008). Moreover, data about changes in carbohydrate metabolism in connection to age polyethism of honeybee workers has been studied (Ohashi et al. 1999; Costa and Cruz-Landim 2005).

Just as in other insects, the concentration of glucose seems to be the main signal that affects the rate of carbohydrate metabolism in bees (Meyer-Fernandes et al. 2001; Kim and Rulifson 2004). The main digestive enzymes of carbohydrates present in the alimentary track of adult bees has been studied. The α-amylase that hydrolyses starch contained in pollen (Ohashi et al. 1999) and β-glucosidase (Pontoh and Low 2002) are secreted from the hypopharyngeal glands of honeybee workers. High activity of sucrase—that degrades sucrose in nectar to glucose and fructose—was found in salivary glands. This enzyme may comprise 50% of all proteins in this gland (Nishimoto et al. 2001; Kubota et al. 2004).

Glucose is the main energetic substrate for bee tissues. Excess of glucose is moved to the fat body where it is used for synthesis of trehalose or stored in a form of glycogen. When energy demands are high, during flight for example, fast degradation of glycogen by glycogen Phosphorylase and trehalose by trehalase provides glucose, which is delivered through the hemolymph to flight muscle as well as to other tissues (Gmeinbauer and Crailsheim 1993; Becker et al. 1996; Blatt and Roces 2001). Trehalose is the main sugar found in hemolymph of honeybees. Its concentration is very high and varies from 2 mg/mL to 40 mg/mL (see Blatt and Roces 2001). Other sugars found in the hemolymph are glucose and fructose. Their concentrations are relatively low: 15 µg/mL and 7 µg/mL for glucose and fructose, respectively (Leta et al. 1996). According to Panzenböck and Crailsheim (1997), such low hemolymph concentration of glucose and fructose is possible because the honeybee has unlimited access to food rich in simple carbohydrates. A high concentration of trehalose in the hemolymph is beneficial because trehalose, a nonreducing sugar, does not react with amino group of proteins, which could be very dangerous given open circulatory systems of insects. In addition, trehalose is very important as an osmotic factor, which might facilitate sugar transport from the midgut to hemolymph (Blatt and Roces 2001).

There are some data concerning carbohydrate metabolism during the larval stages of bee development (Bishop 1923, 1925; Hepburn et al. 1979; Hrassing and Crailsheim 2005). The changes in carbohydrate profile during development of the drone brood have been described (Lipiński et al. 2008). The date of this paper suggested that they might be the result of modifications of the activity of carbohydrates enzymes occurring at the same time. Unfortunately, there is a lack of information about enzymes participating in carbohydrate metabolism during development of honeybee broods. As it was demonstrated in a review by Hrassingg and Crailsheim (2005), great differences occur in many aspects of drone and worker physiology. Thus, the purpose of this paper is the recognition the activity of glucose-providing enzymes during development of the brood, and comparison of the developmental pattern of carbohydrates enzymes activity between workers and drones in the Carniolan honeybee, *A. m. carnica.*

Kaftanoglu et al. (2010) reared larvae in vitro and found that the weight of larvae and imago workers might be regulated by the amount and composition of a brood's diet. Moreover, the sugar content in the diet of female larvae determines the size and degree of ovariole development and thus the future reproductive success of females (Kaftanoglu et al. 2010). It was observed that presence of glucose and sucrose in larval diets prevented premature pupation of *Psacothea hilaris.* Low concentration of glucose in the hemolymph was a key signal to begin this process (Munyiri and Ishikawa 2005). Although effects of change of glucose concentration in hemolymph in the development of honeybee broods have not yet been studied, it may be assumed that glucose could also be an important signal in developmental growth of honeybee broods. The glucose level in hemolymph is strongly affected by the activity of enzymes studied in this work. Therefore, understanding the changes in the development of their activity may be important.

## Materials and Methods

### Honeybee brood

The honeybee is a social holometabolous insect. There are two sexes: females, that are divided into queen and worker castes; and males called drones (Jay 1963). The complete development of a honeybee occurs in a cell of honeycomb (Jay 1963). The eggs hatch three days after being laid by a queen, and for the first three days after hatching, all larvae are fed with royal jelly, a product of hypopharyngeal and mandibular glands of nurse bees. A change in diet of workers and drone larvae then occurs; they receive a mixture of royal jelly, honey, and pollen up to the moment when the cell is sealed, but queens continue to be fed royal jelly (Babendreier et al. 2004). The sealing of cells by nurse bees occurs on the eighth day of worker development, or two days later in case of drones. For a few hours thereafter, larvae consume the remaining food. Then, they become spinning larvae and their cocoon is formed, straightening up and changing into prepupae. The worker imago emerges from cells 21 days after the queen lays the eggs. In the case of drones, the adult emerges after 24 days (Winston 1987).

The present study investigated a worker and drone brood of *A. m. carnica* collected in the middle of June 2008 from an apiary located 20 km of Olsztyn, northeast Poland. During the 30-minute transportation, honeycombs were wrapped in slightly humid towels to maintain appropriate conditions of temperature (20 °C) and humidity.

Shortly after bringing specimens to the laboratory, the brood was very carefully isolated from honeycombs and separated into developmental stages based on morphological features according to Jay (1962, 1963). Twelve developmental stages were distinguished. Starting from egg hatching, unsealed larvae of both sexes were divided into one- and two-day old (L 1/2), three- (L3), and four-day old (L4). Brood in sealed cells were divided into L6/L7 larvae (six-day old worker larvae or seven-day old drone larvae), spinning-stage larvae (L8), prepupae (PP), pupae with white (P1), white-pink (P2), pink (P3), brown eyes and yellow trunk (P4), and with black eyes and body (P5). Workers and drones newly emerged from cells (A) were studied as well. Isolated individuals were washed and carefully dried on filtration paper. Analytical samples were made. Each sample contained three individuals from a given stage except L1/2, L3, and L4 stages, where one sample contained 30 individuals from the L1/2 stage, 20 individuals from the L3 stage, and 10 individuals from the L4 stage. The samples were weighed and immediately frozen in liquid nitrogen and stored at -70 °C until analysis. The elapsed time from providing combs in the laboratory to freezing the samples lasted was about 40–60 min.

### Preparation of extracts from brood

The samples were homogenized in a glass Potter homogenizer on an ice bath with 2.5 mL cool 0.9% NaCl (1:10 w/v). Homogenates were centrifuged for 15 min at 1000 x g at 4 °C. In the supernatants, activity of enzymes and concentration of protein were determined. The protein content was measured by the Bradford method (1976).

### Determination of enzymatic activity

The activity of α-amylase was determined using the Caraway method (1959) using starch as the substrate. The incubation mixtures contained 50 µL supernatant, 0.85 mL 0.2 M acetate buffer (pH 6.8), and 0.1 mL 1% starch solution (Zoltowska et. al 2007). The incubation lasted 120 minutes at 37 °C.

Amylase activity was expressed in international units (U). The activities of glucoamylase and disaccharidases (trehalase and sucrase) were assessed by measuring the amount of glucose released by these enzymes from their specific substrates (Dahlqvist 1968). 50 µL of extract and 0.1 mL 1% solution of glycogen from oyster (Sigma-Aldrich, www.sigmaaldrich.com) in the case of glucoamylase, or 0.1 mL 0.5 mM trehalose or sucrose solution in the case of trehalase and sucrase, respectively, was added to 0.35 mL 0.2 M acetate buffer (pH 4.8) (Zoltowska et. al 2007). The incubation lasted 60 minutes at 37 °C. Concentration of glucose was determined using an enzymatic kit (Cormay, www.pzcormay.pl). The activity of enzymes was expressed by the amount of µmol of glucose per mg of protein. Total activity of glycogen Phosphorylase was measured based on its degradation of glycogen according to Meyer-Fernandes et al. (2001). The assay mixture contained: 50 µL of extract, 40 mM potassium phosphate buffer (pH 7.0), 5 mM imidazole, 2 mM NaEDTA, 1.4 mM dithioyhreitol, 5 mM magnesium acetate, 2 mM AMP, 0.6 mM NADP^+^, 2 mg/mL glycogen, 4 U phosphoglucomutase, and 0.8 U glucose-6-phosphate dehydrogenase (all chemicals from Sigma-Aldrich). Controls without adding enzymes, glycogen, and AMP were done to correct results by measuring the activity of endogegenous of NADP^+^-dehydrogenase in extracts. The activity of enzymes was expressed by the amount of µmol of glucoso-1-phosphate formed during 15 min incubation at 37 °C, estimated using 1 mg of protein.

**Table 1.**
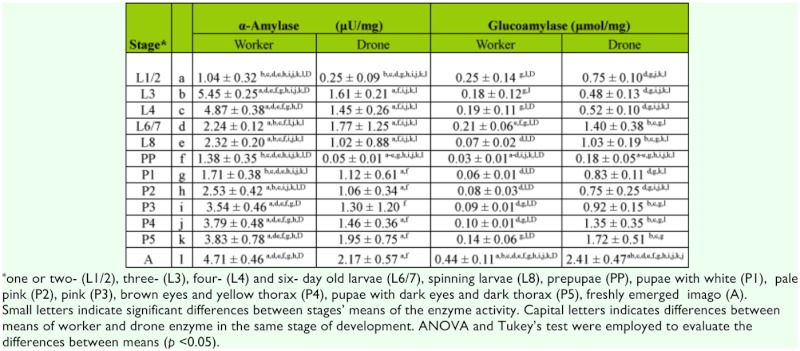
The activity of amylases during development of *Apis mellifera carnica.*

### Statistical analysis

The experiment was done in three replicates, each containing five samples of every studied stage. The ANOVA and Tukey's test were employed to evaluate the differences between means of the enzymatic activities (*p* < 0.05).

## Results

In all developmental stages, polysaccharides were degraded by enzymes in a pathway of hydrolysis (amylase and glucoamylase) and phosphorolysis (glycogen Phosphorylase). It was observed that in brood of both sexes, the activity of glycogen Phosphorylase was two orders higher than amylolytic enzymes. Generally, the activity of α-amylase was low in both sexes of bees, and glucoamylase also had weaker activity in workers than in drones (Table 1).

**Table 2.**
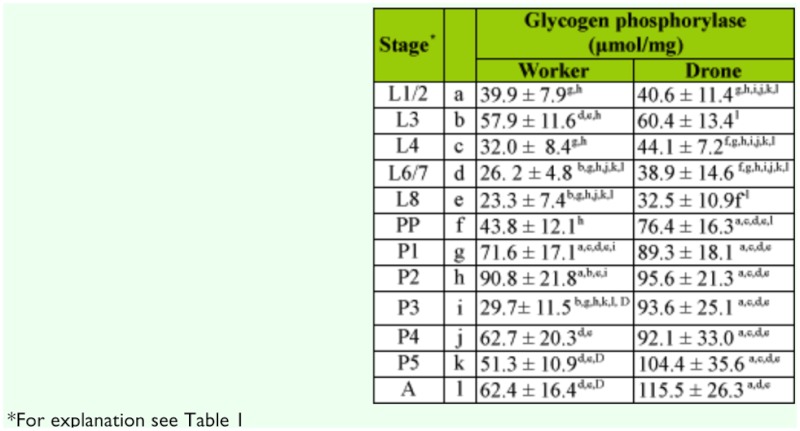
The activity of glycogen Phosphorylase during ontogenesis of *Apis mellifera carnica.*

**Table 3.**
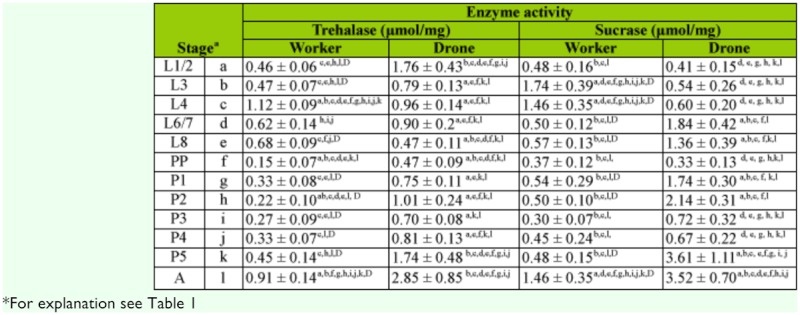
The activity of disaccharidases in ontogenesis of honeybee.

The activity of enzymes in drones and workers differed relative to their developmental stages. These differences were significant for the majority of α-glycosidases (Tables 1 and 3). α-amylase and sucrase had higher activity in unsealed worker larvae than in drone larvae. After the prepupal stage all enzymes were more active in drones than in workers with the exception of amylase. The activity of glucoamylase was many times higher in drones than in workers during all stages of development (Table 1). A similar situation occurred in the case of glycogen Phosphorylase, but differences of means were significant only for P3, P5, and A (Table 2). In the sealed brood of workers, activities of disaccharidases (trehalase and sucrase) were at a similar level, but in newly emerged workers the activity of sucrase was higher than trehalase (Table 3). On the contrary, the activity of trehalase was higher than sucrase in younger drone larvae. Sucrase had considerably higher activity than trehalase in drones in the middle (L8 to P2) and final (P5 and A) developmental stages (Table 3).

Common features of developmental changes in the activities of enzymes in workers and drones were that (i) hydrolases had the lowest activity in prepupae stage, (ii) glycogen Phosphorylase was less active in the spinning larva, and (iii) the activity of most enzymes was significantly higher in the oldest pupae and imago than in earlier stages (Tables 1–3).

Soon before the end of metamorphosis, the activity of all α-glucosidases started to increase. The activity of glucoamylase, trehalase, and sucrase in newly emerged workers was 2- and 3-fold higher than in the oldest pupa stage (P5). In drones, the activity of glucoamylase and trehalase also increased in this period, although it was significantly lower in drones (by around 2 times) than in workers (2–3 times) (Tables 1 and 3, see A vs. P5). The activity of amylase and sucrase in drones, P5, and imago stages was not different (Tables 1 and 3).

## Discussion

Our results showed that phosphorolysis is the main metabolic pathway of endogenic stores of glycogen in both sexes of honeybee broods. Both amylolytic enzymes in workers broods can contribute only in a small degree, due to their low activity in the degradation of glycogen. However, the high activity of glucoamylase in drones may be an important factor that effectively supports action of glycogen Phosphorylase.

In insects, metabolic pathways of the most important sugars, glycogen and trehalose, are connected though common intermediates, such as glucose-1-phosphate and glucose-6-phosphate (Becker et al. 1996). They found that changes of enzymatic activities responsible for degradation of endogenic saccharides (glycogen Phosphorylase and trehalase) were similar in the development of both genders. In feeding (unsealed) larvae, the activities were negatively correlated, which enabled accumulation of reserves of glycogen in the fat body; however, in later periods in non-feeding stages they were positively correlated, demonstrating provision of a suitable amount of glucose to tissues. The results of Becker et al. (1996) are in agreement with a previous study concerning the content of sugars in the development of drone larvae; shortly before sealing, drone larvae had the highest (117 mg/g body mass) content of glycogen (Lipiński et al. 2008). This is in agreement with the observed decrease of glycogen Phosphorylase activity at this particular time. Additionally, accumulation of glycogen may be facilitated by enhanced activity of glycogen synthase in unsealing larvae, which was stated by Vardanis (1967). It is worthwhile to note that this enzyme synthesized glycogen with extra-long external chains. This structure of honeybee glycogen may accelerate action of glycogen Phosphorylase, which prefers longer glucose chains (Hers 1976), and hence provide more substrates for synthesis of trehalose. In drone larvae, the trehalose concentration reached its maximum later than glycogen, during the spinning larval stage. It was supposed that conversion of glycogen into trehalose occurs at that time (Lipiński et al. 2008). Our results are in accordance with that suggestion, and indeed an increase of the activity of glycogen Phosphorylase was observed in L8 larvae.

Schmolz et al. (2005) established that a decrease in the rate of metabolism in larvae of the honeybee occurs after sealing the cells. This observation was reflected in our studies as a periodic decrease of the activity of most enzymes, which was especially visible in prepupae of both genders. Additionally, significant increases in the rate of metabolism were observed in non-feeding pupae, with the exception of the observed decrease in body mass (Hepburn et al. 1979; Schmolz et al. 2005). Our finding of a gradual increase in the activity of enzymes of sugar metabolism in pupae of both sexes is in agreement with the above data. This increase was especially significant in the final stages of metamorphosis of honeybee broods.

Development time of drones is three days longer than workers (Jay 1963). Newly emerging drones have 1.8–2.6 times higher body mass than workers have, and higher body mass is related to higher nutritional demand (Hrassingg and Crailsheim 2005). According to data reported by the above-mentioned authors, the total amount of carbohydrates required for raising workers is 59.4 mg and 98.2 mg for drones. Interestingly, this finding was not fully confirmed in higher activity of enzymes hydrolyzing sugars observed in unsealed drone larvae (Tables 1–3). Enzymes degrading the main carbohydrates of royal jelly and honey-pollen mix, sucrase, and α-amylase had higher activity in unsealed workers than in drone larvae. In drone larvae, only the activity of glucoamylase was significantly higher. It is probable that this enzyme, with broad substrate specificity in relation to α-glucosides (Sauer et al. 2000), can substitute other α-glucosidases and has a more important role in ontogenesis of the drone (Tables 1–3).

The energy needed for larval development of the honeybee from all castes was determined based on calorimetric studies (Schmolz et al. 2005). Significantly higher rates of metabolism were observed in drones than in workers in all developmental stages; our results are consistent with these data. Almost all enzymes have higher activity in drones than workers. This situation becomes especially visible beginning from the prepupae stage (Tables 1–3). The exception was α-amylase, the less significant enzyme for drones, because starch is not the energetic fuel used for their flight (Hrassingg et al. 2005).

It should be highlighted that our enzymes of focus have similar activity profiles during the development process in broods of both workers and drones (Tables 1–3). Their relatively high activity in the youngest larvae, significant decrease in activity in the prepupae stage, and strong increase in final developmental period is evidence of this similarity. Similar results were obtained in previous studies of 19 hydrolases belonging to three classes of enzymes: esterases, peptidases and glucosidases in drone larva of *A. m. carnica.* A decrease in the activity of α-glucosidases was observed in prepupae stage followed by increase in the final stages of development (Zółtowska et al. 2007). This increase may suggest that honeybee pupae are preparing for adult life. The newly emerged imago should be metabolically adapted to fulfill its tasks, and young workers have to be soon ready to function as nurse bees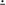 Its role is to process collected pollen and nectar, form royal jelly for feeding younger larvae, prepare and provide the honey-pollen mix to older larvae, feed drones and queens, and maintain homeostasis in beehive. Enzymatic adaptation to age polyethism was especially well documented in the case of the development of the hypopharyngeal glands of workers (Ohashi et al. 1999). In drones, adaptation relies on preparation for effective usage of food prepared by workers and accumulation of energy reserves for reproduction aims (gamete production and mating flights) (Crailsheim 1998). Occurrence of highly active sucrase, trehalase, and glucoamylase would seem very helpful for this.

Proper metabolism of broods, including sugars—the most important of energetic substrates—has biological value for the adult honeybees (Wheeler 1996; Hrassingg and Crailsheim 2005; Hoover et al. 2006). In addition to improving understanding of honeybee biology, this work may give some useful information for apiculture sciences pointing toward the possibility of using some form of carbohydrates for bees rearing, especially for *in vitro* cultures.
